# Analysis of factors influencing the job satisfaction of medical staff in tertiary public hospitals, China: A cross-sectional study

**DOI:** 10.3389/fpsyg.2023.1048146

**Published:** 2023-02-02

**Authors:** Xiang Shi, Dekai Xiong, Xingmin Zhang, Miaomiao Han, Liu Liu, Jinian Wang

**Affiliations:** ^1^School of Health Management, Anhui Medical University, Hefei, Anhui, China; ^2^Department of Scientific Research, The First Affiliated Hospital of Anhui University of Traditional Chinese Medicine, Hefei, Anhui, China; ^3^Department of Education, The First Affiliated Hospital of Anhui Medical University, Hefei, Anhui, China

**Keywords:** COVID-19, job satisfaction, medical staff, China, cross-sectional

## Abstract

**Introduction:**

Since the outbreak of the novel coronavirus pneumonia (COVID-19), China has entered normalization phase of its epidemic prevention and control measures that emphasizes ‘precise prevention and control,’ ‘dynamic zeroing’, and ‘universal vaccination’. However, medical staff continue to face physical and mental stress. The present study aimed to investigate the job satisfaction of medical staff in China, as well as any associated factors.

**Methods:**

2,258 medical staff completed a questionnaire specially designed for this study. Independent samples *t*-tests, one-way analysis of variance, and binary logistic regression were used to analyze associated factors.

**Results:**

Overall, 48.4% of the participants expressed satisfaction with their job; the highest-scoring dimension was interpersonal relationships (3.83 ± 0.73), while the lowest scoring dimension was salary and benefits (3.13 ± 0.94). The logistic regression model indicated that job satisfaction among medical staff is associated with being aged 40–49 years [odds ratio (OR) = 2.416] or > 50 years (OR = 2.440), having an above-undergraduate education level (OR = 1.857), holding a position other than doctor [i.e., nurse (OR = 3.696) or ‘other’ (OR = 2.423)], having a higher income (OR = 1.369), and having fewer monthly overtime shifts (OR = 0.735–0.543). Less than half of the medical staff expressed satisfaction with their job, indicating that the overall level is not high.

**Discussion:**

This research enriches the study of medical workers’ job satisfaction during periods when epidemic prevention and control has become familiar and routine. To improve medical workers’ job satisfaction, administrators should seek to enhance medical staff’s remuneration, reduce their work pressure, and meet their needs (where reasonable).

## 1. Introduction

The 2019 outbreak of novel coronavirus pneumonia (COVID-19) has, as a result of its rapid spread and the difficulties regarding its prevention and control, caused significant harm to economic and social development (Zhao et al., 2020). To address this issue, the Chinese government has implemented measures such as ‘precise prevention and control’, ‘dynamic zeroing’, and ‘universal vaccination’ (Geng and Zhang, 2022) and, through the joint efforts of medical staff and the national people, the epidemic is now under better control (Zhu et al., 2021). Even so, concern for the physical and mental health of medical staff in a pandemic still be taken seriously.

Compared to other occupations, medical staff experience higher levels of stress, anxiety, and depression (Kunzler et al., 2020). This may be because their daily work involves not only providing medical care, but also providing psychological comfort and spiritual support. Moreover, the growing stress associated with their work means medical staff have a higher risk of sudden death and other diseases (Gao et al., 2021); a study found that medical staff have a higher risk of cardiovascular disease than the general population (Yu et al., 2021). Further, Zhou et al. (2019) found, in a survey of 136 tertiary public hospitals in China, that only 48.22% of respondents were satisfied with their job, and had the lowest satisfaction scores for remuneration among 8 items of job satisfaction. Many factors negatively impact medical workers’ job satisfaction, including heavy workloads and job stress, low pay levels, high job risks, and social media coverage of negative medical events (Sun et al., 2018). COVID-19 appeared suddenly, and spread rapidly over a short period of time. Consequently, medical staff involved in combatting this pandemic experienced a higher workload, a greater risk of infection, and heavier than usual physical and mental stress. Epidemiological studies of the 2002 outbreak of severe acute respiratory syndrome have shown that pandemic situations can increase medical workers’ risk of depressive symptoms, along with their risk of post-traumatic stress disorder, anxiety, panic, and even death wish (Liu et al., 2012). Similarly, a study of medical workers conducted during the COVID-19 outbreak found that some workers were experiencing psychological and mental-health problems such as insomnia, anxiety, obsessive–compulsive symptoms, and depression (Zhang et al., 2020).

Currently, medical staff satisfaction surveys tend to concentrate on primary and secondary health care facilities, possibly because of the relatively high benefits offered by staff in higher education public hospitals (Zhou et al., 2018). In fact, tertiary medical personnel are subject to more work pressure and tasks. In 2020, there were 2,996 tertiary public hospitals in China, accounting for 8.46% of the total number of health institutions of all types; however, providing 54.12% of outpatient services and 51.07% of inpatient services, which is much higher than other levels of health institutions (Ministry of Health [MoH], 2020). Thus, there is an urgent need to focus on the job satisfaction of medical personnel in tertiary hospitals.

Job satisfaction is an important element and indicator of hospital management, and can be used to assess hospital employees’ cognition and evaluation of their work, mental state, and emotional health (Nantsupawat et al., 2017). This includes management and leadership (Coomber and Barriball, 2007), remuneration packages (Bjork et al., 2007), workload and work stress (Shamian and El-Jardali, 2007), social relationships (Ahmad and Oranye, 2010), social support (Ramoo et al., 2013) and so on. Further, some studies have shown that health-care workers’ job satisfaction is highly correlated with their turnover intention (Xue et al., 2022), service quality, and service efficiency (Aiken et al., 2012). Health-care workers with higher job satisfaction provide better health-care service and show a stronger sense of belonging to their hospital (Judge et al., 2017). In contrast, lower job satisfaction decreases motivation and enthusiasm, and also has a negative impact on patient satisfaction (Meng et al., 2018). Additionally, under the current situation of COVID-19 prevention and control, medical staff have been found to provide better care to their patients if they perceive themselves as being treated fairly and with respect (Fang X. H. et al., 2021). Thus, it is necessary to determine the psychological health of medical staff during the COVID-19 pandemic. However, Fang F. et al. (2021) found that the prevention and control of the pandemic significantly increased the overall workload of medical staff. Through the SCL90 survey, Hu et al. (2021) found that there was heterogeneity in the psychological status of primary care staff and that ensuring rest, psychological training, and strengthening exercise could help promote their psychological health. Zha et al. (2021) investigated the level of burnout among medical staff during the pandemic and found a correlation with age, occupation, and marital status. In addition, a survey on the sleep status of medical staff found that the incidence of sleep quality problems was as high as 40.35%, significantly higher than the national average (Xiu-Juan et al., 2021). Nevertheless, there has been relatively little research on medical workers’ job satisfaction and the factors that may have reduced their job satisfaction during the pandemic.

The present study focusses on the medical staff of tertiary public hospitals in Anhui Province, investigating their current job satisfaction and exploring factors associated with their level of job satisfaction. This study may enrich research of job satisfaction during the current situation of COVID-19 prevention and control, and may represent an important reference for improving medical staff’s motivation and enthusiasm, and for the development of management strategies for hospitals.

## 2. Methods

### 2.1. Study design and data collection

We performed, from July 27 to September 27, 2021, a survey of medical workers in Anhui Province. Anhui Province is located in the east-central China, and has the 3rd largest population in the region (National Statistical Office, 2020). We chose Anhui Province for the following reasons: (1) As a nation-regional medical center (Development and Reform Commission, 2019), Anhui has abundant medical resources and a medium level of economic development; (2) immediately following the outbreak of COVID-19 in Wuhan, Anhui initiated its emergency response mechanism, and emphasized the provision of logistical support to medical personnel; and (3) Hospitals and health institutions in Anhui have expressed support to this investigation.

For this study, we divided Anhui Province into central, southern, and northern regions, based on the characteristics of these regions and their levels of economic development. We randomly selected five tertiary hospitals located in ‘central’ Anhui (the central region contained the most hospitals), and four tertiary hospitals from each of the ‘southern’ and ‘northern’ regions. We contacted the administrative staff of each hospital with the assistance of Health Commission of Anhui Province to facilitate the survey. They were asked to introduce the purpose and significance of the study to the participants in staff meetings and to voluntarily encourage their participation in the survey. An electronic questionnaire was sent to participants *via* WeChat, which is the most popular social media service in China. Upon accessing the questionnaire, participants were asked to sign an informed consent form, and were given five minutes to complete the questionnaire. We used the formula:


(1) $$N=\mathrm{deff}\frac{\mu^{2}p(1-p)}{\delta^{2}}$$

Using the 4th Chinese doctor’s investigation data, we learned that 51.49% of medical personnel are satisfied with the professional environment (Yu et al., 2018), *μ* = 1.96, an allowable error is 15%. *δ* = 0.5149 × 0.15. Considering that 10% of the questionnaires were invalid, 13 hospitals needed a total of 2,322 samples. Once the number of questionnaires met our sample requirements, we stopped distributing the electronic questionnaires. After eliminating 64 invalid questionnaires, 2,258 valid questionnaires remained, with a valid return rate of 97.24%.

The inclusion criteria were as follows: (1) being a medical worker with over one year of work experience; (2) willing to provide informed consent and voluntarily participate in this survey. The following exclusion criteria were applied: (1) being a non-employee of the hospital (including medical staff in training or individuals performing internships or trainee-ships); (2) unwilling to complete the questionnaire. Questionnaires with three or more items with missing values were considered invalid; for those with 1–2 questions with missing values, the missing values were replaced by the average score of the relevant items in the sample.

### 2.2. Demographic variables and job characteristics

The demographic items measured in our study were as follows: gender, age, years of work experience, education level, marital status, specialization of the participants’ professional departments, occupation, whether the participants had authorized strength, professional title, monthly income (CNY), number of monthly overtime shifts, whether the participants had job-hopped, and whether the participants were members of dual-income families. In this study, job-hopped was when a person left his or her previous workplace to find another job at the current health institution, and a dual-income family was defined as a family in which both the husband and wife have a stable source of income and, thus, more disposable income, and the authorized strength in China was a way of appointment by the government to pay the salary.

### 2.3. Measurement of job satisfaction

Based on consultation of the Minnesota Satisfaction Questionnaire and the Ask-Form Employee Satisfaction questionnaire developed by Graen et al. (1968), we created a questionnaire to assess job satisfaction. The scale contained 34 items and seven dimensions: compensation and benefits, job content, career development, organizational management, work environment, interpersonal relationships, and hospital culture. The overall Cronbach’s α coefficient for the questionnaire was 0.962, and the coefficient result from a Kaiser-Meyer-Olkin test was 0.937, indicating that the revised questionnaire had good reliability. Each item was scored using a five-point Likert scale (1 = ‘very dissatisfied’, 2 = ‘dissatisfied’, 3 = ‘satisfied’, 4 = ‘very satisfied’, and 5 = ‘extremely satisfied’; score range: 34–170); the higher the score, the higher the respondent’s satisfaction level.

### 2.4. Statistical analysis

IBM SPSS Statistics for Windows, version 20.0 (IBM Corp., Armonk, NY, United States), was used to analyze and process the data. The survey respondents’ basic information was statistically described using frequency and composition ratio, their level of job satisfaction was measured using means and standard deviations (*M* ± SD), and the factors associated with their level of job satisfaction were analyzed using independent samples *t*-tests, one-way analysis of variance, and binary logistic regression. Overall, job satisfaction was set as the dependent variable; each participant’s overall level of job satisfaction was calculated based on their overall mean score for the questionnaire. Participants with a score of <3.5 were allocated to the ‘dissatisfied’ group (0), while all other participants were allocated to the ‘satisfied’ group (1). After combining the results of the univariate analysis, statistically significant independent variables were entered into a binary logistic regression model, with job satisfaction as the dependent variable. The significance level was set at *α* = 0.05.

### 2.5. Ethics approval and consent to participate

The study was approved by the Clinical Medical Research Ethics Committee of the First Affiliated Hospital of Anhui Medical University (PJ2021-10-11) and the study team obtained informed consent from all participants. All methods were in accordance with the 1964 Helsinki declaration and its later amendments or comparable ethical standards. The participants were also well informed that completion of the questionnaire signified their informed consent.

## 3. Results

### 3.1. Demographic characteristics of participants

As shown in Table 1, of the 2,258 respondents most were female (70.7%); the largest age group was 30–39 years (48.1%), and the largest work-experience group was ‘≥10 years’ (42.7%). Most participants were undergraduate students (59.7%), with graduate students being the second-largest group (32.4%). In addition, most participants were married (78.6%), 40.7% were physicians, and 43.7% were nursing staff. The largest group regarding professional department was the internal medicine department (34.8%), and 36.6% were established medical staff. Most participants had junior (39.6%) or intermediate titles (44.8%), and most had a monthly income of less than 8,000 (CNY; 58.6%); further, most performed less than five overtime shifts a month (62.8%). Some of the study participants had job-hopped (19.8%), and 58.9% were in dual-income households.

**Table 1:** Participants’ demographic characteristics (*N* = 2,258).

Item	Category	*N*	*%*
Gender	Male	662	29.3
	Female	1,596	70.7
Age	<30	747	33.1
	30–39	1,087	48.1
	40–49	294	13.0
	>50	130	5.8
Work experience (years)	≤5	736	32.6
	6–9	558	24.7
	≥10	964	42.7
Education	Undergraduate below	180	8.0
	Undergraduate	1,347	59.7
	Undergraduate above	731	32.4
Marital	Married	1775	78.6
	Others	483	21.4
Nature of the department	Internal	786	34.8
	Surgery	657	29.1
	Others	815	36.1
Occupation	Doctor	920	40.7
	Nurse	986	43.7
	Others	352	15.6
Whether had authorized strength	Yes	827	36.6
	No	1,431	63.6
Professional title	Junior	894	39.6
	Intermediate	1,012	44.8
	Senior	352	15.6
Monthly income (CNY)	<7,000	1,324	58.6
	>7,000	934	41.4
Monthly overtime (times)	≤5	1,419	62.8
	6–9	442	19.6
	≥10	397	17.6
Whether job-hopped	Yes	446	19.8
	No	1812	80.2
Whether dual-income family	Yes	927	41.1
	No	1,331	58.9
Overall	Satisfied	1,093	48.4
	Dissatisfied	1,165	51.6

### 3.2. Job satisfaction score for each dimension

The satisfaction scores for each dimension are shown in Table 2. Ranking of the items from highest to lowest score proceeds as follows: interpersonal relationships (3.83 ± 0.73), hospital culture (3.62 ± 0.79), job content (3.61 ± 0.73), work environment (3.60 ± 0.75), career development (3.54 ± 0.78), organizational management (3.51 ± 0.82), and compensation and benefits (3.13 ± 0.94). All dimensions had a mean score of ≥3, showing that the participants had above-average satisfaction for all dimensions.

**Table 2:** Job satisfaction score for each dimension.

Item	Job satisfaction *n* (%)					Job satisfaction score (*M* ± SD)
	Very dissatisfied	Dissatisfied	Satisfied	Very satisfied	Extremely satisfied	
Compensation and benefits	58 (2.5)	252 (11.2)	986 (43.7)	697 (30.9)	265 (11.7)	3.13 ± 0.94
Job content	15 (0.6)	38 (1.7)	635 (28.1)	1,189 (52.7)	381 (16.9)	3.61 ± 0.73
Career development	14 (0.6)	61 (2.7)	726 (32.2)	1,112 (49.2)	345 (15.3)	3.54 ± 0.78
Organizational management	25 (1.1)	86 (3.8)	833 (36.9)	1,001 (44.3)	313 (13.9)	3.51 ± 0.82
Work environment	12 (0.5)	56 (2.5)	652 (28.9)	1,173 (51.9)	365 (16.2)	3.60 ± 0.75
Interpersonal relationships	10 (0.4)	32 (1.4)	447 (19.9)	1,252 (55.4)	517 (22.9)	3.83 ± 0.73
Hospital culture	19 (0.8)	43 (1.9)	747 (33.1)	1,064 (47.1)	385 (17.1)	3.62 ± 0.79

### 3.3. Single factor analysis of job satisfaction

We compared job-satisfaction scores across participants with different demographic characteristics, and found statistically significant differences for gender, age, work experience, education, occupation, whether the participants had authorized strength, average monthly income, and average number of monthly overtime shifts (*p* < 0.05). These results are shown in Table 3.

**Table 3:** Single-factor analysis of participants’ job satisfaction (*M* ± SD).

Item	Category	Scores	*t/F*	*p-*value
Gender	Male	3.35 ± 0.70	−8.136	<0.001
	Female	3.62 ± 0.72		
Age	<30	3.55 ± 0.70	3.909	0.008
	30–39	3.49 ± 0.73		
	40–49	3.62 ± 0.76		
	>50	3.66 ± 0.66		
Work experience (years)	≤5	3.50 ± 0.68	3.425	0.033
	6–9	3.52 ± 0.71		
	≥10	3.58 ± 0.75		
Education	Undergraduate below	3.60 ± 0.66	12.140	<0.001
	Undergraduate	3.59 ± 0.75		
	Undergraduate above	3.44 ± 0.68		
Marital	Married	3.55 ± 0.73	0.985	0.325
	Others	3.51 ± 0.70		
Nature of the department	Internal	3.52 ± 0.77	0.969	0.380
	Surgery	3.53 ± 0.71		
	Others	3.56 ± 0.69		
Occupation	Doctor	3.29 ± 0.67	100.649	<0.001
	Nurse	3.74 ± 0.72		
	Others	3.61 ± 0.65		
Whether had authorized strength	Yes	3.48 ± 0.72	−2.881	0.004
	No	3.57 ± 0.75		
Professional title	Junior	3.52 ± 0.70	0.422	0.656
	Intermediate	3.55 ± 0.74		
	Senior	3.54 ± 0.72		
Monthly income (CNY)	<7,000	3.49 ± 0.71	−3.948	<0.001
	>7,000	3.61 ± 073		
Monthly overtime (times)	≤5	3.65 ± 0.71	50.194	<0.001
	6–9	3.40 ± 0.69		
	≥10	3.29 ± 0.71		
Whether job-hopped	Yes	3.40 ± 0.72	−4.665	0.225
	No	3.57 ± 0.72		
Whether dual-income family	Yes	3.47 ± 0.74	−3.544	0.695
	No	3.58 ± 0.71		

### 3.4. Binary logistic regression for job satisfaction

Job satisfaction was found to have significant relationships with gender, age, work experience, education level, job category, monthly income, and average number of overtime shifts per month, respectively (*p* < 0.05). Job satisfaction was 1.266-times higher among female participants than male participants [OR = 1.266; 95% CI = (1.005, 1.594); *p* < 0.05]; when compared to participants aged under 29 years, those aged 40–49 years had 2.416-times higher job satisfaction [OR = 2.416; 95%CI = (1.57, 3.719); *p* < 0.05] and those aged ≥50 years had 2.44-times higher job satisfaction [OR = 2.44; 95% CI = (1.426, 4.175); *p* < 0.05]; participants with graduate degrees were 1.857-times [OR = 1.857; 95%CI = (1.218, 2.831); *p* < 0.05] more satisfied with their jobs than those with a qualification lower than a bachelor’s degree; when compared to doctors, nurses had 3.696-times higher job satisfaction [OR = 1.264; 95% CI = (2.749, 4.97); *p* < 0.05] and other medical professionals had 2.423-times higher job satisfaction [OR = 2.423; 95%CI = (1.827, 3.213); *p* < 0.05]; participants earning 8,000–17,000 (CNY) per month were 1.369-times [OR = 1.369; 95%CI = (1.157, 1.619); *p* < 0.05] more satisfied than those earning less than 8,000 (CNY) per month; participants who performed an average of 6–9 and ≥ 10 overtime shifts per month were 0.735-times [OR = 0.735; 95% CI = (0.584, 0.925]; *p* < 0.05) and 0.543-times [OR = 0.543; 95% CI = (0.423, 0.697); *p* < 0.05], respectively, less satisfied than those who performed less than five overtime shifts per month (Table 4).

**Table 4:** Binary logistic regression for job satisfaction.

Item	Category	*B*	*P*-value	OR	95% CI
Gender	Male				
	Female	0.236	0.045	1.266	(1.005,1.594)
Age	<30				
	30–39	0.090	0.529	1.094	(0.827,1.447)
	40–49	0.882	<0.001	2.416	(1.570,3.719)
	>50	0.892	<0.001	2.440	(1.426,4.175)
Work experience (years)	≤5				
	6–9	−0.139	0.327	0.871	(0.660, 1.149)
	≥10	−0.257	0.134	0.774	(0.553, 1.083)
Education	Undergraduate below				
	Undergraduate	0.234	0.18	1.264	(0.897, 1.781)
	Undergraduate above	0.619	0.004	1.857	(1.218, 2.831)
Occupation	Doctor				
	Nurse	1.307	<0.001	3.696	(2.749, 4.97)
	Others	0.885	<0.001	2.423	(1.827, 3.213)
Whether had authorized strength	Yes				
	No	−0.041	0.731	0.96	(0.761, 1.211)
Monthly Income (CNY)	≤7,000				
	>7,000	0.314	<0.001	1.369	(1.157, 1.619)
Monthly overtime (times)	≤5				
	6–9	−0.308	0.009	0.735	(0.584, 0.925)
	≥10	−0.611	<0.001	0.543	(0.423, 0.697)

## 4. Discussion

In this study, we investigated the job satisfaction of medical staff in Anhui Province at a point when COVID-19 prevention and control efforts had become familiar and routine. The average satisfaction score for the 2,258 medical workers who participated in our survey was 3.54 (±0.72), and only 48.4% of the sample expressed satisfaction; this is far from the 80% required by the Hospital Management Evaluation Guidelines developed by the Chinese Ministry of Health (Tao et al., 2021). However, when compared with a satisfaction survey conducted before the epidemic (Zhou et al., 2018), our research shows a relative increase in the satisfaction of health-care workers; a possible reason for this is that the health-care workers felt that fighting the epidemic was a common goal, and they consequently united to help each other.

Interpersonal relationships, hospital culture, and job content were the highest-scoring items in terms of satisfaction. This may be explained by the unique role played by medical staff in the current pandemic context, and the fact that their hard work is widely recognized and the government and society have shown consideration for their safety. On September 8, 2020, at the National Commendation Ceremony to Combat the New Pneumonia Epidemic, medical staff were praised, being referred to as the ‘most beautiful angels’ and the ‘loveliest people of the new era’ (Xinhua News Agency, 2020). Moreover, on February 7, 2020, the National Health and Wellness Commission issued the ‘Notice on Efforts to Protect Frontline Medical Staff and Their Families’, which featured recommendations for protecting frontline medical staff’s quality of life, psychological health, and safety, and also featured humanism-based considerations (National Health Commission of the People’s Republic of China, 2020). Furthermore, the government fully covers the cost of COVID-19 treatment and vaccination for frontline medical staff. The Chinese government’s responsible attitude in this regard has greatly reduced the concerns of patients’ families and promoted good cooperation and mutual trust between patients and medical staff (State Council of China, 2020). The subsequent increase in patient satisfaction has contributed to the professional fulfillment and job satisfaction of medical staff (Szecsenyi et al., 2011).

Through binary logistic regression, we found that income level is a positive predictor of satisfaction, and that number of overtime shifts is a negative factor. Analysis of each dimension showed that salary and welfare is the area for which medical staff have the lowest level of satisfaction. This was consistent with the overall results of this study. There are two possible reasons salary level is the primary factor affecting the job satisfaction of medical staff. On one hand, medical staff are frontline workers in terms of the prevention and control of the COVID-19 epidemic, and this role is associated with several risks; even today, at a time when protective measures have been implemented, frontline workers continue to face a higher risk of infection (Ning et al., 2020). Such risks can be compounded by difficulties balancing their high-intensity workload with their family life. On the other hand, although in times of severe epidemics medical staff may be given allowances depending on their workload and the difficulty of their role, during times when epidemic prevention and control have normalized duties associated with combatting the epidemic are included in medical staff’s routine work; this means medical workers may be required to perform more work without receiving corresponding compensation.

We discovered that female medical staff are more satisfied than male medical staff, which is contrary to the findings of Wang and Wan (2022). In fact, Chinese parents generally help women to reduce the burden of child care, which facilitates Chinese women in having more time and energy to focus on their own work career development (Xiong et al., 2022). At the same time, in order to reciprocate the support of the female community (e.g., having more vacation time), Chinese women are more motivated and engaged in their work, which subjectively contributes to increased satisfaction (Rolf et al., 2018). Meanwhile, consistent with the findings of similar studies, job satisfaction showed a general ‘U’-shaped trend across age groups; medical staff aged 30–39 years showed the lowest job satisfaction, which may be explained by the greater life-and work-related burden and economic pressure experienced by this age group (Wu et al., 2019).

According to our logistic regression, the factors associated with medical staff’s job satisfaction are professional characteristics; specifically, educational background, occupation, monthly income, and average number of monthly overtime shifts. Thus, the satisfaction level of medical staff varies across different occupational characteristics. Regarding educational background, we found that the higher a medical worker’s education level, the higher his/her job satisfaction. This contrasts with the findings of Yu et al. (2020), who found, in a 2020 analysis of medical staff in Wuhan, that the more educated the medical staff, the less satisfied they were with their work. A probable reason for this discrepancy is that, after the epidemic moved into a phase of normalization, there was a reduction in the proportion of people with high education levels who were performing epidemic-prevention work at the grass-roots level. The higher one’s education level, the more likely he/she is to hold an important position and, thus, the higher his/her compensation. Regarding medical staff with lower education levels, differentiated incentives may be an important method of improving their job satisfaction (Feifei et al., 2022).

Nursing staff and other medical technical administrators showed higher job satisfaction than doctors. This finding may be associated with the nature of epidemic prevention, including the day-to-day work, as doctors, as the primary authorities in diagnoses of diseases and formulation of treatment plans, must quickly and accurately respond to medical conditions and carefully manage their relationships with patients. Further, doctors have higher occupational risks than many other positions, with over 10% of doctors routinely facing five high-level occupational risks (risk of infection, risk of violence, environmental factors, medical error, bias; Li et al., 2014).

The results of this survey provide a reference for improving the job satisfaction of medical and nursing staff. Firstly, this can be achieved by increasing these staff’s overall income level and implementing incentive protection policies that make medical staff’s performance during the pandemic an important element in the evaluation of titles and employment. In this study, when the medical staff’s income was raised, their enthusiasm for work increased. Second, the working and resting conditions of medical staff should be guaranteed, occupational protection facilities and equipment should be strengthened, and the quality of life must be well protected. Work tasks and staff time must be reasonably arranged, and the deployment of medical forces and the arrangement of work shifts must be dynamically adjusted. Additionally, health tests of medical staff should be correctly performed, and the rest time should be reasonably arranged according to their physical state. These measures can, to a certain extent, alleviate the decrease in satisfaction brought about by the increased workload.

### 4.1. Limitations

This study has several limitations. First, the survey was limited to medical staff in Anhui Province, China, meaning that the findings do not reflect the full spectrum of job satisfaction among medical staff across the country. For nationwide validation, the study should be further expanded, taking into account the policies and economic and social development of different regions. Second, since we employed a cross-sectional study design, the time sequence between exposure (possible correlates) and outcome (job satisfaction) cannot be considered. Time-based data could be collected in future studies to further explore the factors influencing job satisfaction. In addition, the study omitted some intrinsic factors of job satisfaction, including sense of calling (Tung et al., 2020), productivity expectations (Randolph, 2005), responsiveness (Ludwig-Beymer et al., 2022), the opportunity to use one’s abilities (Goetz et al., 2012), etc. Inclusion of intrinsic factors in the study design. Positive factors such as the opportunity to use one’s abilities had the most positive impact on job satisfaction, and sense of calling was associated with reductions in both job dissatisfaction and burnout.

## 5. Conclusion

In this study, we investigated job satisfaction among medical staff, and its associated factors, at a time when epidemic prevention measures in Chinese tertiary hospitals had become familiar and routine. Our study showed that gender, age, education level, occupation, monthly income, and average number of monthly overtime shifts can impact job satisfaction. We suggest that hospital management bodies improve medical staff’s satisfaction by improving their treatment, reducing their work pressure, and providing differentiated incentives.

## Data availability statement

The original contributions presented in the study are included in the article/supplementary material, further inquiries can be directed to the corresponding authors.

## Ethics statement

The studies involving human participants were reviewed and approved by the Clinical Medical Research Ethics Committee of the First Affiliated Hospital of Anhui Medical University (PJ2021-10-11). The patients/participants provided their written informed consent to participate in this study.

## Author contributions

XS reviewed the topic related literature, wrote the first draft, analyzed the data, and revised the manuscript. MH, DX, and XZ performed the study coordination and data collection. LL performed study concept, data collection, and study supervision. JW performed the study concept and design, obtained funding and carried out study supervision, revision of the manuscript, and guarantor for the study. All authors checked, interpreted results and approved the final version, contributing to the article equally.

## Funding

This work was supported by Sichuan Provincial Education Department Key Research Base of Humanities and Social Sciences Sichuan Hospital Management and Development Research Center Grant: An Empirical Study on Humanistic Care System of Hospital Health Care Workers in the Context of New Coronary Pneumonia (grant no. SCYG2020-01), and Doctoral Research Project, The First Affiliated Hospital of Anhui Medical University (Subject No. BSKY2022067).

## Conflict of interest

The authors declare that the research was conducted in the absence of any commercial or financial relationships that could be construed as a potential conflict of interest.

## Publisher’s note

All claims expressed in this article are solely those of the authors and do not necessarily represent those of their affiliated organizations, or those of the publisher, the editors and the reviewers. Any product that may be evaluated in this article, or claim that may be made by its manufacturer, is not guaranteed or endorsed by the publisher.
